# Dietary Fructose Reduction Improves Markers of Cardiovascular Disease Risk in Hispanic-American Adolescents with NAFLD

**DOI:** 10.3390/nu6083187

**Published:** 2014-08-08

**Authors:** Ran Jin, Jean A. Welsh, Ngoc-Anh Le, Jeffrey Holzberg, Puneet Sharma, Diego R. Martin, Miriam B. Vos

**Affiliations:** 1 Department of Pediatrics, School of Medicine, Emory University, 2015 Uppergate Drive NE, Atlanta, GA 30322, USA; E-Mails: rjin@emory.edu (R.J.); jean.a.welsh@emory.edu (J.A.W.); jholzberg@gmail.com (J.H.); 2 Children’s Healthcare of Atlanta, Atlanta, GA 30329, USA; 3 Biomarker Core Laboratory, Atlanta Veterans Affairs Medical Center, Decatur, GA 30033, USA; E-Mail: anhleatcsl@gmail.com; 4 Department of Radiology, Emory University, Atlanta, GA 30322, USA; E-Mails: puneets@email.arizona.edu (P.S.); dmartin@radiology.arizona.edu (D.R.M.)

**Keywords:** nonalcoholic fatty liver disease, hepatic steatosis, fructose, sugar, cardiovascular risk, obesity, children and adolescents

## Abstract

Nonalcoholic fatty liver disease (NAFLD) is now thought to be the most common liver disease worldwide. Cardiovascular complications are a leading cause of mortality in NAFLD. Fructose, a common nutrient in the westernized diet, has been reported to be associated with increased cardiovascular risk, but its impact on adolescents with NAFLD is not well understood. We designed a 4-week randomized, controlled, double-blinded beverage intervention study. Twenty-four overweight Hispanic-American adolescents who had hepatic fat >8% on imaging and who were regular consumers of sweet beverages were enrolled and randomized to calorie-matched study-provided fructose only or glucose only beverages. After 4 weeks, there was no significant change in hepatic fat or body weight in either group. In the glucose beverage group there was significantly improved adipose insulin sensitivity, high sensitivity C-reactive protein (hs-CRP), and low-density lipoprotein (LDL) oxidation. These findings demonstrate that reduction of fructose improves several important factors related to cardiovascular disease despite a lack of measurable improvement in hepatic steatosis. Reducing dietary fructose may be an effective intervention to blunt atherosclerosis progression among NAFLD patients and should be evaluated in longer term clinical trials.

## 1. Introduction

Nonalcoholic fatty liver disease (NAFLD) has become a major cause of chronic liver disease worldwide. Global NAFLD prevalence estimates range from 2.8% to 46%, with increased risk in patients with type 2 diabetes mellitus [1,2,3]. The rising number of children with NAFLD is of considerable public health concern because they may have a more aggressive, earlier onset form of the disease. Estimates of NAFLD in the general pediatric population range from 3% to 11% [4,5,6]. In the United States, Mexican-American children appear to have the highest prevalence compared to Caucasians and African Americans [7]. NAFLD can lead to end stage liver disease but also is closely associated with cardiovascular disease (CVD) [8,9,10]. It is suspected to exacerbate the pathogenesis of CVD through the systemic release of inflammatory and oxidative-stress mediators and through insulin resistance and atherogenic dyslipidemia [11]. Although longitudinal studies in pediatric patients with NAFLD are not available to examine long-term CVD risk, a growing body of evidence suggests that adolescents with NAFLD already have increased subclinical atherosclerosis [12,13,14]. In view of the high prevalence of NAFLD among adolescents and the strong association between NAFLD and CVD, exploring early prevention strategies through diet and lifestyle modification and developing effective treatment options are clearly needed.

One of the dietary culprits in the promotion of NAFLD in susceptible adolescents could be high consumption of fructose. Sugars containing fructose are widely used as sweeteners in beverages and processed foods and consumption is much higher today compared to past decades [15,16,17,18,19]. Recent data have shown that fructose intake represents more than 12% of daily calories consumed by U.S. adolescents (primarily from sweet beverages) [19], which exceeds the current recommendations [20,21]. In both animal and short-term human feeding studies, fructose increases hepatic fat accumulation and plasma triglyceride concentration in part through an increase in *de novo* lipogenesis [22,23,24]. Fructose also increases oxidative damage by reducing antioxidant defenses and enhancing the production of reactive oxygen species (ROS) [25,26,27]. This combination of lipid overload and oxidative stress makes fructose suspect in the development and progression of NAFLD [28,29]. Furthermore, fructose-induced dyslipidemia, insulin resistance, and oxidative damage could specifically contribute to the increased CVD risk seen in NAFLD [30]. However, direct evidence showing the benefits of fructose restriction on hepatic steatosis or CVD risk in NAFLD is still lacking, especially in adolescents, a group characterized by both high prevalence of NAFLD and high intake of fructose [6,19].

In the current study, we recruited overweight Hispanic-American adolescents with frequent consumption of sweet beverages and elevated hepatic fat (>8%) as measured by state-of-the-art magnetic resonance spectroscopy (MRS) methodology [31]. In a calorie-matched, randomized, controlled study, we examined whether hepatic steatosis and associated cardiovascular risk factors would be improved after 4 weeks of substitution of usual high fructose containing beverages with study-provided glucose only beverages.

## 2. Materials and Methods

### 2.1. Subjects and Study Design

**Figure 1:**
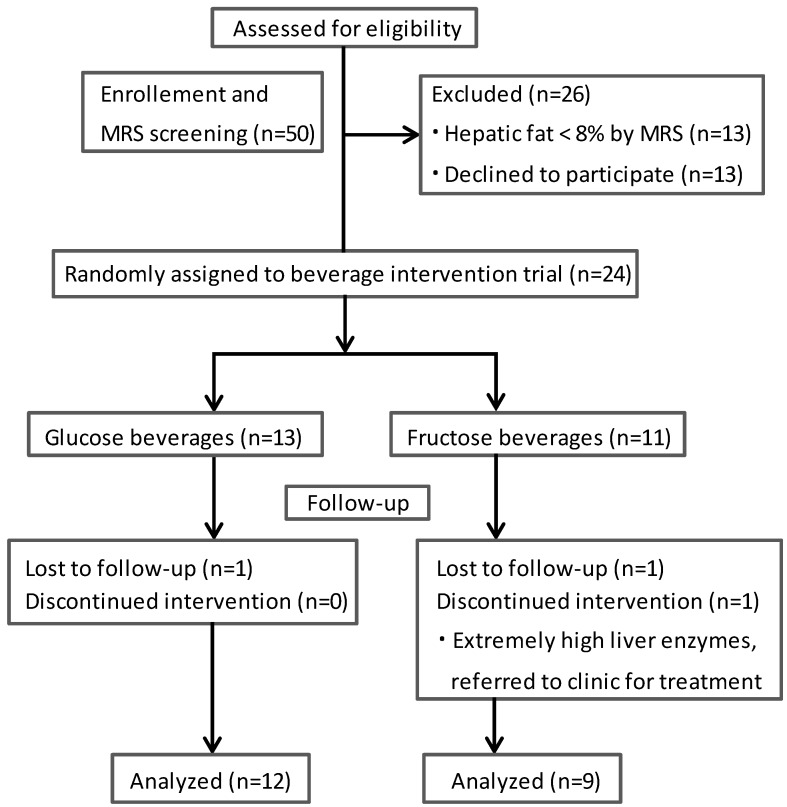
Study design for the randomized controlled beverage trial.

This was a 4-week, double-blinded, randomized, controlled intervention study (registered at clinicaltrials.gov, NCT01188083). The study design is summarized in Figure 1. Overweight (BMI *z*-score ≥ 85th percentile) adolescents were recruited from pediatric clinics at Emory Children’s Center and from nearby community centers through flyers and presentations at community events. Eligibility criteria included self-identification as Hispanic, ages 11–18 years; BMI ≥ 85th percentile for age and gender; and average self-reported consumption of at least 3 servings of sweet beverages (equivalent to 24 fl oz) per day. Sweet beverages were defined as drinks sweetened with added sugars (e.g., sodas, sweet tea, sport drinks, flavored drinks) or naturally sweet beverages (e.g., 100% fruit juice), but did not include artificially-sweetened drinks. Although 100% juice is not always included in assessment for sugar consumption, we included it because the fructose content of juice is high [32] and there is little evidence to suggest that it would not have the same effect as fructose from sodas or other sweetened beverages. Exclusion criteria included pregnancy; known liver diseases; diabetes or fasting glucose ≥126 mg/dL; renal insufficiency (creatinine >2 mg/dL); chronic systemic disease requiring daily medication; acute illness within past 2 weeks prior to enrollment (defined by fever >100.4 F); currently attempting weight gain or weight loss; and anti-oxidant therapy or supplement within 4 weeks prior to enrollment.

All recruited participants underwent MRS to quantify their hepatic fat; subjects with hepatic fat >8% were randomized within 1 week of the baseline MRS to either study-provided fructose (considered as fructose continuation) or glucose (considered as fructose reduction) beverage groups. Follow-up visits were completed at 2 and 4 weeks after randomization. The interim visit at 2 weeks was intended to assess safety and to promote compliance and was not a primary outcome. Prior to each study visit, subjects were required to fast for at least 12 h and fasting blood samples were collected for the laboratory measurements between 7 and 9 am on the next morning. Initially, a total of 24 adolescents agreed to participate in this randomized controlled 4-week beverage trial. At the 2-week visit, one subject’s serum alanine aminotransferase (ALT) increased to >10 times the upper limit of normal and participation was terminated for safety concerns. Two subjects declined to return after the 2 week visit and dropped out. The remaining 21 subjects successfully completed the study protocol.

During the 4-week study, participants were instructed to drink 3 servings (8 fl oz bottles) of study-provided beverages each day. The study beverages contained 33 g of sugar (standard amount of sugar in a typical soda) in the form of either glucose or fructose and were matched for color and flavoring (Power Brands, Beverly Hills, CA, USA). Each subject was given a sufficient supply of study beverages to take home or beverages were delivered directly to their house by the research coordinator. Participants and investigators were blinded as to the contents of the drinks. No other sugar-containing beverages were allowed during the study period. Subjects were requested not to change their diet pattern and physical activity. Compliance was monitored through daily drink logs, return of empty beverage bottles at each study visit, and weekly phone calls from the study coordinator; and it did not differ between groups. The study protocol was approved by the Emory University and Children’s Healthcare of Atlanta IRBs and written informed consent (parental consent obtained for subjects <18 years) and assent (when applicable) were obtained for each subject prior to participation in the study.

### 2.2. Determination of Hepatic Fat

Hepatic fat was assessed by MRS using our previously described methods [31]. Briefly, we used a rapid 15 s acquisition technique obtained during a single breath hold. The sequence is constructed from five concatenated echoes using a fixed set of echo times (TE) (12, 24, 36, 48, and 72 ms), with each echo having a repetition time (TR) = 3000 ms, voxel = 3 × 3 × 3 cm^3^, 1024 points, and 1200 Hz bandwidth. The acquisition was repeated three times for reproducibility. Data were exported off-line for automatic processing with in-house software (Matlab, Mathworks, Natick, MA, USA). Water and lipid magnitude spectra were analyzed by determining the area under the curve (AUC) corresponding to a user-defined frequency range surrounding the corresponding water/lipid peaks (water peak: 4.6 ppm; lipid peak: 1.3, 2.0 ppm). The integrated magnitude signals at each TE were fit to exponential T2 decay curves, whereby the equilibrium signal (M_0_) and the relaxation rate (R2 = 1/T2) were determined by least-squares regression approximation. Using M_0_ for water and lipid, the T2-corrected hepatic lipid fraction was calculated from: % Hepatic Lipid = M_0lipid_/(M_0lipid_ + M_0water_).

### 2.3. Laboratory Analyses

Fasting blood samples were collected into EDTA-coated tubes and plasma was separated immediately. Plasma samples were protected from light and transported on ice to the laboratory for further processing (within 4 h). All lipid measurements were performed by the Emory Lipid Research Laboratory using AU480 chemistry analyzer (Beckman Coulter, LaBrea, CA, USA). Total cholesterol and triglycerides were measured by enzymatic method using reagents from Beckman (Beckman Diagnostics, Fullerton, CA, USA). Low-density lipoprotein (LDL) and high-density lipoprotein (HDL) were measured by homogeneous enzymatic assay (Sekisui Diagnostics, Exton, PA, USA). Free fatty acid (FFA) and glucose was quantified by colorimetric method (Sekisui Diagnostics, Exton, PA, USA). Insulin and high sensitivity C-reactive protein (hs-CRP) were assessed using immunoturbidometric method (Sekisui Diagnostics, Exton, PA, USA).

Plasma level of oxidized LDL (oxLDL) was quantified using Mercodia human oxLDL ELISA kit (Winston-Salem, NC, USA) which is based on the 4E6 antibody against oxidized apolipoprotein B (apoB). Baseline and week 4 serum plasminogen activator inhibitor-1 (PAI-1) concentrations were assessed using an ELISA kit (abcam, Cambridge, MA, USA). Very low density lipoprotein (VLDL) particle numbers and sizes were measured by nuclear magnetic resonance (NMR) spectroscopy using a 400-MHz proton analyzer (LipoScience Inc., Raleigh, NC, USA) as described elsewhere [33]. Serum ALT and aspartate aminotransferase (AST) were measured by the Emory University Hospital Medical Laboratory.

### 2.4. Insulin Resistance Index

Insulin resistance was assessed by the homeostasis model of assessment—Insulin resistance (HOMA-IR) and the newly defined adipose insulin resistance (IR) index [34]. HOMA-IR was calculated by glucose (mmol/L) × insulin (mU/L)/22.5 at the fasting state, and adipose IR index was calculated as fasting FFA (mEq/L) × insulin (mU/L).

### 2.5. Measurement of Oxidative Stress

The *ex vivo* LDL oxidative susceptibility assay was performed using the method previously described by Esterbauer *et al.* [35]. Freshly collected plasma samples (2 mL) were adjusted with high density solution of NaBr containing 0.1% EDTA to density of 1.21 g/mL and subjected to ultracentrifugation at 39,000 rpm for 48 h (15 °C) using the 50.4 Ti rotor (Beckman Instruments, Palo Alto, CA, USA). The supernate (*d* < 1.21 g/mL) was fractionated into VLDL, LDL, and HDL by fast protein liquid chromatography (FPLC, Thermo Scientific, Waltham, MA, USA) [36]. The oxidative susceptibility of LDL was assessed by continuous monitoring of the formation of conjugated dienes during the lipid peroxidation at the absorbance of 234 nm with the DU530 spectrophotometer (Beckman Coulter, LaBrea, CA, USA). Briefly, 40 μg LDL cholesterol in 0.01 M PBS without EDTA was exposed to 9 μM Cu_2_SO_4_ and the kinetics of lipid peroxidation was monitored for a total of 300 min at the interval of 1 min at room temperature. Lag time, an indicator of oxidative susceptibility, was defined graphically as the time (min) to the initiation of oxidation. LDL with longer lag phase would be more resistant to oxidative modification and is described as having low oxidative susceptibility. To minimize inter-assay variability, LDL preparations from each participant collected at baseline and week 4 were assayed in the same batch.

### 2.6. Statistical Analyses

Statistical analyses were performed using SPSS (version 17.0, SPSS Inc., Chicago, IL, USA). Results in the tables were reported as mean (standard error) unless indicated otherwise. Statistical significance was considered as *p* ≤ 0.05. Data were examined for normality and equal variance prior to any analyses. Baseline differences between two study beverage groups were examined using the Mann-Whitney test and the gender difference was evaluated with the Fisher’s Exact test. Changes from baseline to week 4 within each beverage group were compared by Wilcoxon test, and the differences in % change over the 4 weeks between two beverage groups were assessed by conducting the Mann-Whitney test. We based our sample size calculation on the assumption that an absolute change of ±3% in hepatic fat would be clinically important. Using the mean hepatic fat and standard deviation from our pilot data in a similar cohort, we estimated that greater than 90% study power would be achieved by recruiting 6 subjects in each beverage group with p value set at 0.05.

## 3. Results

The baseline parameters of participants randomized to the two beverage groups are presented in Table 1. There were no significant differences in age, sex, body weight, liver measurement, lipid profile, and glycemic status between the two groups.

**Table 1:** Baseline characteristics of participants enrolled in the 4-week intervention trial.

Parameters	Fructose (*n* = 9)	Glucose (*n* = 12)
Age (years)	14.2 (0.88)	13.0 (0.71)
Male, *n* (%)	3 (33.3)	8 (66.7)
Body weight (kg)	82.3 (5.62)	82.0 (4.27)
BMI *z*-score	2.25 (0.19)	2.15 (0.09)
Hepatic fat (%)	14.5 (1.79)	14.0 (1.77)
ALT (U/L)	33.0 (6.74)	32.7 (5.24)
AST (U/L)	32.4 (3.06)	33.8 (2.11)
Triglycerides (mmol/L)	1.77 (0.39)	1.78 (0.20)
Cholesterol (mmol/L)	4.34 (0.24)	4.40 (0.34)
LDL (mmol/L)	2.79 (0.27)	2.87 (0.29)
HDL (mmol/L)	1.19 (0.08)	1.12 (0.06)
FFA (mmol/L)	0.97 (0.08)	1.11 (0.14)
Glucose (mmol/L)	5.53 (0.28)	5.04 (0.37)
Insulin (mU/L)	30.4 (4.29)	36.3 (9.29)
hs-CRP (mg/L)	6.78 (3.16)	5.21 (1.34)

Values are presented as means (standard error); BMI, body mass index; ALT, alanine aminotransferase; AST, aspartate aminotransferase; LDL, low-density lipoprotein; HDL, high-density lipoprotein; FFA, free fatty acid; hs-CRP, high sensitivity C-reactive protein.

As shown in Table 2, after the 4-week intervention, adolescents receiving study-provided glucose beverages (considered as fructose reduction) had significant improvement in plasma hs-CRP (*p* = 0.028), adipose IR index (*p* = 0.004), and plasma FFA (*p* = 0.027) as compared to baseline. In addition, they had a significant reduction in circulating oxLDL levels (*p* = 0.034). The susceptibility of LDL to Cu^2+^-induced oxidative modification was significantly improved as demonstrated by a 2-fold increase in LDL lag time (*p* = 0.010). In the group receiving fructose beverages (considered as fructose continuation), there were no significant changes in plasma FFA, hs-CRP, adipose IR index, oxLDL levels or the *ex vivo* oxidative susceptibility of LDL. For both groups, no significant changes were observed in body weight, hepatic fat, liver enzymes, fasting TG, and PAI-1 levels after 4 weeks.

**Table 2:** Liver measurement, lipid profile, glycemic status, and inflammation and oxidation status at baseline and 4 weeks after consumption of study-provided fructose or glucose beverages in adolescents with hepatic steatosis.

Parameters	Fructose (*n* = 9)			Glucose (*n* = 12)		
	Baseline	Week 4	*p*-Value	Baseline	Week 4	*p*-Value
Body weight (kg)	82.3 (5.62)	83.0 (5.86)	0.150	82.0 (4.27)	82.5 (4.17)	0.419
Hepatic fat (%)	14.5 (1.79)	13.6 (1.83)	0.314	14.0 (1.77)	13.8 (1.92)	0.814
ALT (U/L)	33.0 (6.74)	33.4 (4.41)	0.678	32.7 (5.24)	33.8 (5.69)	0.562
AST (U/L)	32.4 (3.06)	33.3 (3.34)	0.953	33.8 (2.11)	32.8 (2.10)	0.531
Triglycerides (mmol/L)	1.77 (0.39)	1.15 (0.12)	0.139	1.78 (0.20)	1.72 (0.23)	0.754
FFA (mEq/L)	0.97 (0.08)	0.90 (0.10)	0.767	**1.11 (0.14)**	**0.78 (0.07)**	**0.027**
Glucose (mmol/L)	5.53 (0.28)	5.20 (0.24)	0.374	5.01 (0.37)	5.20 (0.26)	0.638
Insulin (mU/L)	30.4 (4.29)	45.1 (9.78)	0.260	36.3 (9.29)	29.5 (5.28)	0.859
Adipose IR	28.6 (3.78)	36.1 (5.59)	0.214	**34.6 (7.86)**	**21.4 (3.82)**	**0.004**
HOMA-IR	7.38 (0.97)	10.7 (2.68)	0.441	8.40 (2.38)	6.44 (0.99)	0.754
hs-CRP (mg/L)	6.78 (3.12)	7.06 (2.36)	0.477	**5.21 (1.34)**	**3.99 (1.09)**	**0.028**
LDL lag time (min)	18.6 (4.55)	25.8 (6.13)	0.084	**18.5 (3.66)**	**37.1 (8.39)**	**0.010**
Oxidized LDL (mU/L)	8.82 (0.98)	7.88 (1.11)	0.515	**8.49 (0.97)**	**7.06 (0.96)**	**0.034**
PAI-1 (ng/ml)	47.3 (2.59)	49.5 (2.34)	0.594	51.0 (1.90)	51.2 (2.39)	0.594

Values are presented as means (standard error); ALT, alanine aminotransferase; AST, aspartate aminotransferase; FFA, free fatty acid; Adipose IR, adipose insulin resistance, calculated as fasting FFA (mmol/L) × insulin (mU/L); HOMA-IR, homeostatic model assessment for insulin resistance index, calculated as fasting glucose (mmol/L) × insulin (mU/L)/22.5; hs-CRP, high sensitivity C-reactive protein; LDL, low-density lipoprotein; PAI-1, Plasminogen activator inhibitor-1. **BOLD** indicates statistical significance.

**Figure 2:**
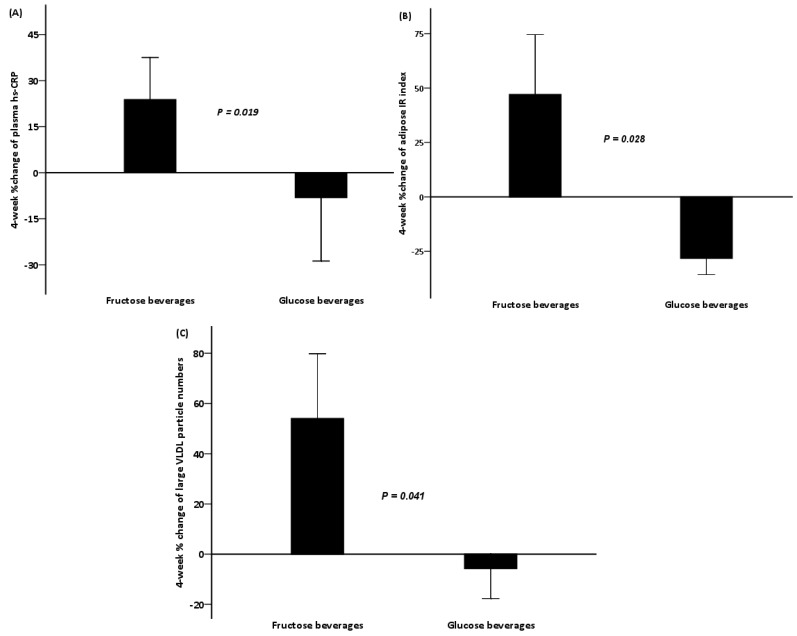
4-week percent changes of plasma hs-CRP (**A**); adipose IR index (**B**); and large VLDL particle numbers (**C**) in both fructose and glucose beverage groups among adolescents with hepatic steatosis. Error bars stand for SE.

We also compared the percent changes in all indicators over 4 weeks between the glucose and fructose beverage groups. Adolescents who were randomized to glucose beverages had significant improvements in hs-CRP (mean ± SE: −8.1% ± 20.7% *vs.* 23.8% ± 13.8%, *p* = 0.019) and adipose IR (mean ± SE: −28.1% ± 7.6% *vs.* 47.0% ± 27.6%, *p* = 0.028) as compared to those who consumed calorie-matched fructose beverages (Figure 2A,B). Furthermore, based on NMR spectroscopy, the percent change of large VLDL particles from baseline to week 4 was significant between the two beverage groups (*p* = 0.041), with the particle number increased by 53.9% ± 25.9% (mean ± SE) for the individuals randomized to fructose beverages and decreased by 5.7% ± 11.9% (mean ± SE) in glucose beverage group (Figure 2C).

## 4. Discussion

NAFLD affects almost one-third of overweight adolescents and has long-term health consequences, particularly from CVD. Some have described NAFLD as the hepatic manifestation of metabolic syndrome because of its frequent association with insulin resistance, increased visceral adiposity, and atherogenic dyslipidemia [37]. Even in childhood, patients with NAFLD demonstrate atherosclerotic lesions as measured by increased carotid intima media thickness (cIMT) [14]. Thus, adolescents with NAFLD are believed to be at high risk of future CVD, and studies are needed that examine modifiable influences contributing to increased cardiovascular risk in NAFLD. In this study, we examined Hispanic-American adolescents because of their dramatically increased risk of NAFLD [38,39]. We performed a calorie-matched, randomized, controlled reduction of fructose in order to isolate the effect of the sugar type from calories added by sweet beverages. We found that fructose reduction through replacing usual sweet beverages with glucose-only beverages resulted in an improved cardiometabolic profile, including increased insulin sensitivity, reduced hs-CRP, fewer large VLDL particles and fewer oxidized LDL. Although we did not observe the amelioration in hepatic steatosis, this combination of improvements in regards to cardiovascular risk in a carefully controlled randomized trial indicates that fructose plays a unique role in the increased CVD risk seen in NAFLD patients.

Several possible mechanisms may explain the metabolic improvements that were observed in this study. Fructose consumption has been shown to increase visceral adipose tissue (VAT) in both animal and human studies [28]. Increased VAT acts as a metabolically active tissue that produces numerous inflammatory cytokines such as hs-CRP, tumor necrosis factor alpha (TNF-α), and interleukin-6 (IL-6) and promotes both hepatic and systemic insulin resistance [3]. On the other hand, fructose has been shown to affect the microbiome and increase endotoxin release into the circulation [22,40]. Increased endotoxin release activates the innate immune system and inflammation, subsequently exacerbating insulin resistance [41]. Impaired insulin sensitivity, in combination with increased VAT, would further dysregulate lipid metabolism, resulting in excess FFA flux from the peripheral tissue returning to the liver and thereby increased hepatic secretion of large, more atherogenic VLDL [42,43]. Consistent with these pathways, in our current study, by reducing fructose consumption for 4 weeks, adolescents with hepatic steatosis showed improved inflammation and adipose insulin sensitivity as indicated by hs-CRP and adipose IR index, along with improved FFA levels and large VLDL particle concentrations in the circulation.

Improvement in oxidative stress may also result from reduced fructose ingestion. We have previously shown that oxLDL improves in response to a low fructose diet over 6 months [44]; however, in that experiment, the subjects made multiple changes in their diets. In this trial, we blinded participants and investigators to the assigned study-provided beverages to minimize the chance that other dietary changes were responsible for the effects. We again found a significant decrease in circulating oxLDL levels and expanded on this by testing LDL susceptibility to oxidation by reducing fructose consumption. Previous work has demonstrated that high fructose regimen (10% in drinking water) decreased oxidation resistance of lipoprotein fractions in copper-induced lipoperoxidation in a rat model [45], which was consistent with our new finding in adolescents with hepatic steatosis. This impaired oxidation resistance could possibly reflect the diminished endogenous antioxidant content of the lipoprotein particles and the increased production of free radicals, as shown in animal studies [25,26,27]. Although consistent in animal studies [46], fructose-induced oxidative stress has not been well demonstrated in human trials and needs to be further evaluated.

We also expected that fructose reduction for 4 weeks would improve hepatic steatosis and PAI-1. Hepatic fat has been shown by others to increase after soda consumption over 6 months [47]. A recent pilot study of fructose reduction in adults with NAFLD indicated a decline in intrahepatic fat content along with weight loss after 6 months [48]. In our study, neither group had a change in hepatic steatosis levels. While this disproved our hypothesis, our study was designed to be eucaloric. The fructose reduction associated with loss of hepatic fat in adults was in the setting of weight loss and this might be a requirement for removing excess stored energy in the liver. Further, since hepatic steatosis may be a downstream result of worsened insulin resistance, visceral fat accumulation, and oxidative stress, it could be that a longer study would have resulted in changes in the liver. Recent studies suggested that glucose overfeeding might also increase intrahepatocellular lipids after conversion to fructose in the liver [49,50]. While we did not see an increase in hepatic fat in either group, both glucose and fructose may support continued presence of steatosis. We tested PAI-1 because it is a coagulant factor that has been found to be strongly associated with hepatic steatosis [51,52]. Increased PAI-1 has been proposed as an underlying mechanism potentially responsible for accelerated atherogenesis in NAFLD [11]. Given the proposed relationship between PAI-1 and steatosis, it is consistent that PAI-1 also did not change in either group. It also suggests that PAI-1 is less related to insulin resistance, as it did not change in parallel with the changes in insulin resistance.

There are some limitations of our pilot study. As an outpatient study, we did not control the entire diet in our study subjects over the intervention period. The body weights of the subjects remained stable from baseline to the end of the study, which implies a eucaloric period. We asked subjects to replace their usual consumption of sweet beverages with the study-provided beverages to minimize other changes in the diet. The 4-week study period was brief compared to the years that adolescents typically consume sweet beverages and a longer study would be helpful to confirm the persistence of the improvements and determine if a longer reduction would also improve liver findings. We selected 4 weeks based on other studies [53,54] and in part to improve compliance. We studied a group of Hispanic adolescents because of their disparate risk for NAFLD and future CVD. These findings are critical for this particular group; however they may not be generalizable to NAFLD patients from other ethnic backgrounds. Finally, due to the sample size, sex and puberty differences in response to the fructose reduction could not be examined in our cohort.

## 5. Conclusions

In summary, this double-blind, randomized controlled study comparing glucose beverages to fructose beverages demonstrates that reduction of fructose for 4 weeks in adolescents with hepatic steatosis (consistent with NAFLD) improves adipose insulin sensitivity, inflammation, plasma FFA, and LDL oxidation. These data suggest that treatment strategies targeting fructose reduction would reduce future cardiovascular risk in adolescents with NAFLD. Longer studies of fructose reduction and fructose reduction studies utilizing non-invasive vascular measurements of atherosclerosis will be needed to prove this. In the meantime, given that fructose reduction is an inexpensive and side-effect free intervention for patients with NAFLD, it can be considered as an adjunct to current therapies.

## Support

NIH K23 DK080953(Vos), North American Society of Pediatric Gastroenterology, Hepatology and Nutrition (NASPGHAN) Foundation Nestle Young Investigator Award (Vos), also supported by the National Center for Advancing Translational Sciences of the National Institutes of Health under Award Number UL1TR000454. The content is solely the responsibility of the authors and does not necessarily represent the official views of the National Institutes of Health.
